# Catalogue of Plants in Kentucky

**Published:** 1833

**Authors:** 


### Art. IX. Catalogue of the Plants of Kentucky.

Professor Short has put forth a *prodromus* of the *Flora* of Kentucky, of which a copy is now before us. It presents

444 genera, and 885 species. We take pleasure in seeing the names of many of our early acquaintances, particularly those of the *arbustum terra fertilis*, for the first time arrayed in alphabetical order; but fancy they would appear in greater dignity if scientifically arranged. We are glad to observe that Dr. S. has adopted the genus *Carya* of Nuttall, for our hickories, and separated them from the old genus *Juglans*. Long before Mr. N. established that family, we had observed such differences between the hickories and walnuts of the valley of the Ohio, as excited our astonishment that they should ever have been united under one generic head. Having a similar impression in respect to our box elder and sugar tree, we should have preferred to see the former (*acer negundo*) taken out of the genus of which the latter is the type. Among the medicinal plants of Kentucky, we notice several that are deserving of attention; as *actea alba* (*racemosa*,) *aralia spinosa*, *aristolochia serpentaria*, *arum dracontium*, *a. triphyllum*, *asclepias*, several species, *asarum Canadense*, *cassia Marylandica*, *cicuta maculata*, *conium maculatum*, *cornus florida*, and other species, *datura stramonium*, *dyospyros Virginiana*, *erigeron philadelphicum*, *eupotorium perfoliatum*, *euphorbia*, several species, *gentiana*, more than one species, *geranium maculatum*, *gillenia stipulacia*, *heuchera Americana*, *humulus lupulus*, *hydrastis canadensis*, *liriodendron tulipifera*, *lobelia inflata*, *monarda*, several species, *phytolacca decandra*, *podophyllum peltatum*, *polygala senega*, *prenanthes*, *prunus Virginiana*, *quercus*, the species generally, *rhus*, three or four species, *sabbatia angularis*, *salix*, *spigelia Marylandica*, and *ulmus Americana*.

This is certainly a promising catalogue, and it might be considerably extended. We regret to find so little attention paid, in these latter days, to our medical botany. It ought to present high and peculiar attractions to the physicians of the West, who, in general, have not the means of prosecuting physiological and pathological inquiries, but might engage with the brightest hopes, in pharmaceutic and clinical experiments, on our native plants.

